# Measuring farmers’ sustainable livelihood resilience in the context of poverty alleviation: a case study from Fugong County, China

**DOI:** 10.1057/s41599-023-01575-4

**Published:** 2023-02-27

**Authors:** Yue Sun, Yanhui Wang, Chong Huang, Renhua Tan, Junhao Cai

**Affiliations:** 1 Key Laboratory of 3Dimensional Information Acquisition and Application, Ministry of Education, Capital Normal University, 100048 Beijing, China; 2 State Key Laboratory of Resources and Environmental Information System, Institute of Geographic Sciences and Natural Resources Research, Chinese Academy of Sciences, 100101 Beijing, China; 3 China Urban Construction Design & Research Institute Co. Ltd., 100120 Beijing, China

**Keywords:** Geography, Development studies

## Abstract

In recent years, the sustainable livelihood of farmers has been threatened by various events such as the COVID-19 pandemic, which has significantly impacted efforts to alleviate poverty. Therefore, it is vitally important to increase farmers’ sustainable livelihood resilience to enhance the stability and sustainability of poverty alleviation efforts. In this study, to scientifically measure and analyze farmers’ sustainable livelihood resilience, we designed an analytical framework that captures the characteristics of farmers’ sustainable livelihood resilience from the three dimensions of buffer capacity, self-organization capacity, and learning capacity. We then constructed an index system of farmers’ sustainable livelihood resilience and a cloud-model-based multi-level fuzzy comprehensive evaluation model. Finally, the coupling coordination degree and decision tree methods were used to identify the level of development and relationships among the three abovementioned dimensions of farmers’ sustainable livelihood resilience. A case study from Fugong County, Yunnan Province, China revealed that the spatial and temporal distributions of farmers’ sustainable livelihood resilience were heterogeneous across various regions. Furthermore, the spatial distribution of the coordinated development level of farmers’ sustainable livelihood resilience is similar to that of its overall level because the three dimensions of buffer capacity, self-organization capacity, and learning capacity interact with each other and develop synergistically, and the lack of any one of these affects the overall development of farmers’ sustainable livelihood resilience. In addition, the sustainable livelihood resilience of farmers in various villages is in a state of stable promotion, benign promotion, stagnation, mild recession, severe recession, or chaotic period, indicating a lack of balance in terms of the state of development. However, sustainable livelihood resilience will gradually improve in response to targeted support policies formulated by the national or local governments.

## Introduction

As the COVID-19 pandemic enters its third year, its catastrophic impact on human lives and livelihoods and global efforts to achieve the Sustainable Development Goals (SDGs) are clear. Statistics show that the emergence of COVID-19 resulted in an increase in the global poverty level from 8.3% in 2019 to 9.2% in 2020, with about eight million additional workers falling into poverty. This is the first increase in extreme poverty since 1998 and the largest annual increase since 1990, setting global poverty reduction efforts back by about 3 years. SDG 1 (No Poverty) has been particularly impacted by multiple crises, and the pandemic halted years of progress toward eradicating extreme poverty. In recent years in China, with the country’s layer-by-layer deployment, all rural poor people under the current standard have been lifted out of poverty, as have all 832 poverty-stricken counties, thereby completing the arduous task of eliminating absolute poverty and entering the post-poverty era (Yu et al., 2021). Thus, China’s focus has shifted from poverty alleviation to stabilizing the outcomes of the poverty alleviation program and establishing a long-term mechanism aimed at alleviating relative poverty. However, the COVID-19 pandemic has presented a new challenge, especially in poverty-stricken mountainous areas, where farmers face not only economic, social, and environmental risks, as well as other risks commonly faced by poor areas but also due to illness, education, disability, accident, industrial project failure, unstable employment, and other specific risk factors for returning to poverty and leading to poverty. China is also facing other severe risks and uncertainties as a result of the epidemic, such as various impacts on migrant workers, agricultural production, children’s education, medical treatment for the elderly, and people’s future livelihoods and plans. In this context, poverty-stricken areas are exposed to various disturbances and shocks, which present serious obstacles to the survival and development of poor households, resulting in poor livelihood sustainability and persistent or repeated poverty for farmers. One reason is that the cultivation of farmers’ livelihood resilience has not received sufficient attention (Wu, 2021). However, the key to lifting relatively poor groups out of poverty lies in changing the longstanding low status of farmers in areas with fragile ecologies and livelihoods, effectively improving their sustainable livelihood resilience and improving their ability to deal with risks and withstand external shocks, thereby reducing the likelihood of returning to poverty (Su et al., 2021; Wang et al., 2021). This is the focus of programs aimed at alleviating poverty among poor mountainous households, and it also guides current rural poverty governance work, that is, boosting poverty alleviation and increasing farmers’ income to enhance their sustainable livelihood resilience (He et al., 2020). Resilience first originated in the field of physics and involved the concept of objects returning to their original state after deformation. Scholars such as Holling then applied the concept to ecology (Holling, 1973). With the subsequent deepening of resilience theory and the expansion of its application, it is now possible to study farmers’ sustainable livelihood resilience (Sina et al., 2019; Eadie et al., 2020; Hofmann, 2021; Bui et al., 2021; Pagnani et al., 2021; Quandt, 2018). The COVID-19 pandemic has provided a major setback to sustainable development everywhere, and yet as with other major crises in the past, new ideas have emerged during the pandemic that might help to advance work aimed at achieving the SDG policies by 2030 and beyond. Therefore, introducing resilience theory into research on poverty alleviation and the sustainable livelihoods of households lifted out of poverty, and exploring the livelihood scenarios of farmers from the perspective of resilience can provide new insights and concepts regarding farmers’ sustainable livelihoods.

Some scholars have carried out exploratory research on the description and measurement of resilience systems from different perspectives. In the research framework and measurement method of resilience, the Resilience Alliance proposed the general steps of resilience evaluation (Yang et al., 2021). Numerous scholars have defined and measured resilience in their respective fields from different perspectives such as post-disaster reconstruction (Sina et al., 2019; Eadie et al., 2020), urban resilience (Hofmann, 2021), and tourism destination resilience (Bui et al., 2021), using comprehensive evaluation, scenario simulation, dynamic models, and other methods (He et al., 2021; Feofilovs and Romagnoli, 2021; Zhang et al., 2019). Current research on household livelihood resilience is mainly focused on two areas. One is to analyze the livelihood resilience of farming households from the perspective of the response relationship of farming households’ livelihood status around a key variable that affects their livelihoods (Daniel et al., 2019; Ifejika Speranza, 2013). The other is based on traditional poverty analysis frameworks such as social deprivation, vulnerability analysis, and sustainable livelihoods, indicators are selected from various perspectives of farmers’ livelihoods for a comprehensive evaluation, and the resilience of farmers’ livelihoods is analyzed based on the evaluation results (Nasrnia and Ashktorab, 2021; Lecegui et al., 2022). In terms of the dimensions used to measure and evaluate farmers’ sustainable livelihood resilience, most scholars adopt the perspectives of buffer capacity, adaptation capacity, and self-organization capacity (Zhang et al., 2020; Speranza et al., 2014; Wen et al., 2018; Liu et al., 2020; Li et al., 2022; Fachrista and Suryantini, 2019), while other scholars have measured sustainable livelihood resilience based on income diversity as a result of farmers’ vulnerability and adaptability (Wu et al., 2017; Reddy et al., 2021). For example, Reddy et al. built a multidimensional Farmers’ Distress Index to study how to reduce the distress of farmers from seven dimensions such as exposure to risk, adaptive capacity, and sensitivity. It provides a reference for the sustainable development and resilience of farmers’ livelihoods. However, the index is mainly constructed from the perspective of vulnerability. Although vulnerability and resilience are two closely related concepts, vulnerability pays more attention to the risks and difficulties that farmers may encounter in their lives, while resilience focuses more on coping with risks and the ability to resist difficulties, so we believe that research from the three dimensions of buffer capacity, self-organization capacity, and learning capacity can better reflect the characteristics of resilience. In addition, the household livelihood capital that is commonly used in sustainable livelihood analysis provides a reference point for the measurement and evaluation of resilience systems (Quandt, 2018). Since there is still no more recognized paradigm for measuring resilience, it is still the mainstream perspective to use different empowerment methods to comprehensively evaluate the resilience of farmers’ livelihoods and analyze the factors influencing resilience, types of farmers’ resilience and strategies to improve them, whether from the perspective of comprehensive consideration of farmers’ livelihood factors or from the perspective of accuracy and analyzability of results (Nasrnia and Ashktorab, 2021; Wen et al., 2018). However, if this evaluation method is to continue to be used, it needs to focus on the characteristics of resilience that differ from those applicable to traditional poverty or development research to fully reveal the unique attributes of resilience. Analysis of livelihood resilience starts with the factors influencing resilience, identifies different groups based on those factors, and puts forward suggestions and countermeasures (Nasrnia and Ashktorab, 2021; Wen et al., 2018), but these studies are still mainly based on the qualitative descriptive analysis of results, lacking the combination with quantitative and positioning assessment methods, the important research topic of analyzing the mechanism of action among the components of resilient systems is often neglected, which makes it difficult to guarantee the practicality and relevance of research results.

Therefore, to better evaluate the sustainable livelihood of farmers in the context of poverty alleviation, we further examine the concept of resilience and use the characteristics of resilience as the basis for measuring the ability of farmers to develop sustainable livelihoods in the new era. Promote the integration and linkage of resilience research frameworks with traditional livelihood research frameworks, and conduct quantitative scientific measurement and analysis of farmers’ sustainable livelihood resilience. This is not only a key point to be further explored in the field of farmers’ sustainable livelihood resilience research, but also an important entry point to respond to the current international research hotspots and to improve the contemporary farmers’ sustainable livelihood coping strategies in China. In this context, we selected Fugong County, a once deeply impoverished county in Yunnan Province, China as the study area, and combined the sustainable livelihood research framework and resilience theory to first clarify the expression and description framework of farmers’ sustainable livelihood resilience in the context of poverty alleviation. Then, using the three dimensions of buffer capacity, self-organization capacity, and learning capacity, we constructed a set of indicators and a model to comprehensively evaluate farmers’ sustainable livelihood resilience. Finally, we proposed a method of classifying various types of sustainable livelihood resilience to enable us to analyze the characteristics of spatiotemporal variations in sustainable livelihood resilience of farmers in the study area from 2015 to 2018 and propose appropriate improvement strategies.

## Study area and data

### Study area

**Fig. 1:**
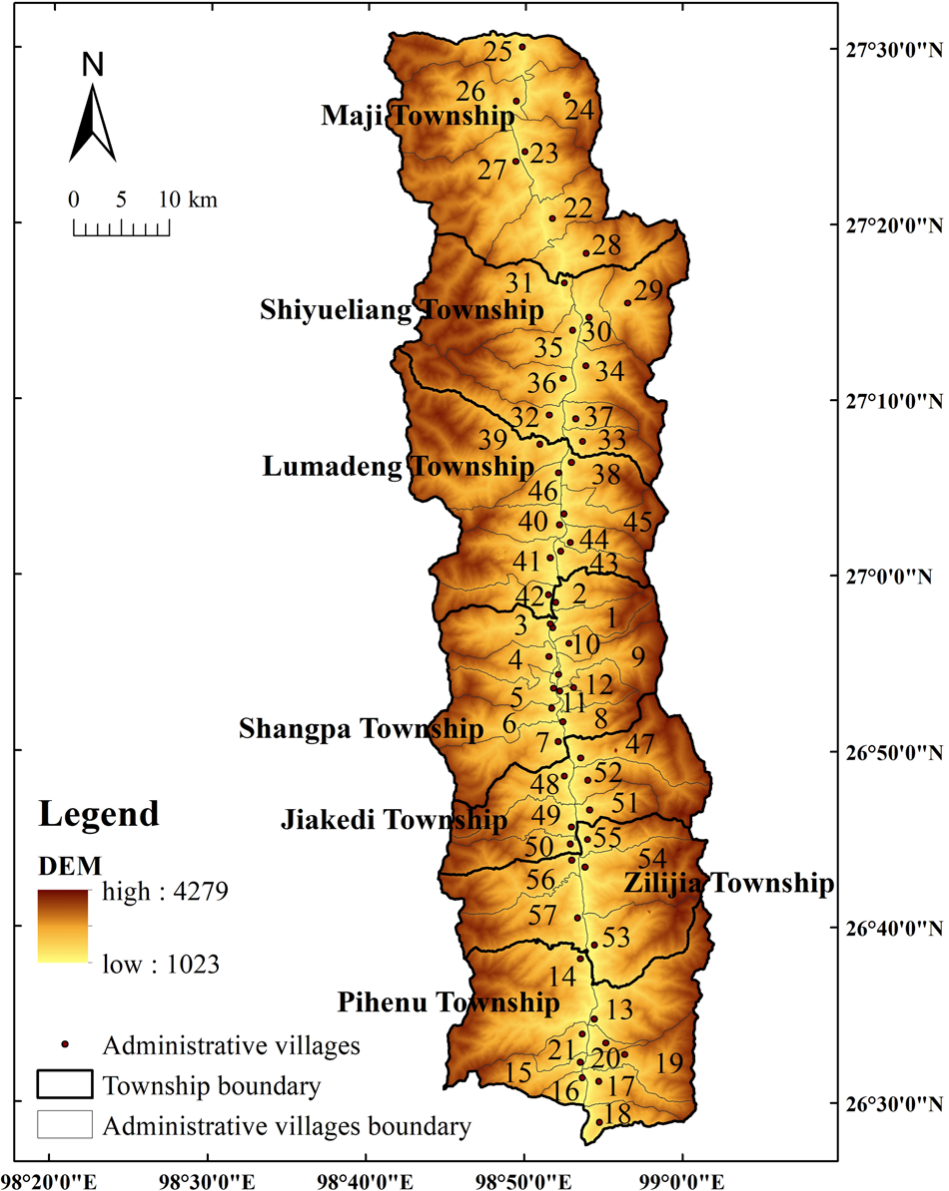
Overview of the study area. The figure shows the geographical location of Fugong County in Yunnan Province in China, including the boundaries of 7 townships and 57 administrative villages in the study area, and the color of the image represents the elevation, with darker colors indicating higher elevations. *Note*: 1. Lazhudi Village 2. Dapuluo Village 3. Shidi Village 4. Zhuminglin Village 5. Latudi Village 6. Guquan Village 7. Mugujia Village 8. Jiziluo Village 9. Shangpa Village 10. Dayou Village 11. Lawu Village 12. Shuangmidi Village 13. Shawa Village 14. Wawa Village 15. Jiajiu Village 16. Tuoping Village 17. Puluo Village 18. Guoke Village 19. Zhiziluo Village 20. Laomudeng Village 21. Miangu Village 22. Qiaodi Village 23. Maji Village 24. Gudang Village 25. Bula Village 26. Mujiajia Village 27. Majimi Village 28. Wangjidu Village 29. Shimendeng Village 30. Lishadi Village 31. Lamadi Village 32. Yaduo Village 33. Zali Village 34. Zuoluodi Village 35. Mi’eluo Village 36. Ziguduo Village 37. Zhiluo Village 38. Chisa Di Village 39. Yaping Village 40. Bajiaduo Village 41. Buladi Village 42. Chihengdi Village 43. Lumadeng Village 44. Lamaluo Village 45. Majiadi Village 46. Watuwa Village 47. Ada Village 48. Weidu Village 49. Jiake Village 50. Liwudi Village 51. Nan’anjian Village 52. Dadake Village 53. Yagu Village 54. Zilijia Village 55. Ekeluo Village 56. Lamujia Village 57. Jinxiugu Village).

We selected Fugong County, Yunnan Province, China as the study area. As shown in Fig. 1, Fugong County, which was once one of the most poverty-stricken counties in China, is a typical mountainous border county. Fugong County has seven townships under its jurisdiction which include 57 administrative villages. The terrain is undulating and is often affected by natural disasters such as landslides. Many villages are located in the mountains, with limited transportation facilities and infrastructure. Thus, this is an appropriate area given the purposes of our study.

### Data and preprocessing

The data used in the study included 30 m digital elevations, 30 m Landsat8 OLI sensor remote sensing imagery, Point-of-Interest data of infrastructure, administrative boundaries, road networks, targeted poverty alleviation data for the period 2015–2018, and socioeconomic data. These data were obtained from the geospatial data cloud, Google Maps, the local poverty alleviation office, and statistical yearbooks. Data cleaning, image preprocessing, projection transformation, georeferencing, and topological relationship inspection were undertaken as required.

## Research methods

This study focused on exploring how to alleviate poverty and achieve long-term sustainable development for farmers. Based on the sustainable livelihood theory, it integrates the concept of resilience and takes the expression, measurement, and analysis of the resilience of farmers’ sustainable livelihood as the main line. First, based on the traditional sustainable livelihood analysis framework, design an expression and description framework that reflects the characteristics of farmers’ sustainable livelihood resilience, and then we constructed an index system of farmers’ sustainable livelihood resilience and a cloud-model-based multi-level fuzzy comprehensive evaluation model based on the three dimensions of buffer capacity, self-organization capacity, and learning capacity.

### Farmers’ sustainable livelihood resilience measurement framework in the context of poverty alleviation

**Fig. 2:**
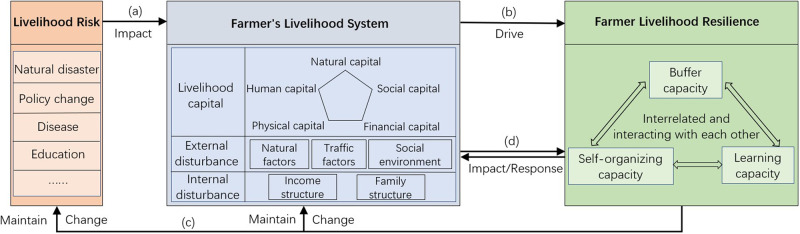
Descriptive framework for farmers’ sustainable livelihood resilience. The figure shows the framework of describing the sustainable livelihood resilience of farm households with livelihood risk, livelihood system, and livelihood resilience as the core elements, and the dynamic process of interactions between the components and within the resilience.

The term “sustainable livelihood” originated from research on poverty by Sen (1982) and Chambers and Conway (1992), and refers to a way of earning a living that enables individuals or households to cope with and recover from stresses and shocks and maintain or enhance their capabilities and assets without destroying natural resources. Following extensive research, the UK Department for International Development proposed the most widely used sustainable livelihood analysis framework (DfID, 1999). Starting from this framework, in the context of a fragile environment and economy, the sustainable livelihood of farmers consists of three elements: livelihood capital, livelihood strategy, and livelihood consequences. However, we believe that the traditional analysis framework for sustainable livelihoods of farmers lacks a comprehensive consideration of the dynamic interactions between farmers and the external environment as well as the internal dynamic of farmers’ livelihood structural elements, which is exactly what a resilient system needs to be paid close attention to in the current context of stable poverty alleviation. Therefore, combining with the definition of sustainable livelihood, this paper extends the traditional sustainable livelihood analysis framework from the perspective of resilience, and constructs a framework for describing the resilience of farmers’ livelihood systems with livelihood risk, livelihood system, and livelihood resilience as the core elements (see Fig. 2). And describes and expresses the sustainable livelihood resilience of farm households from two perspectives: the connotation of the key components in the framework and the analysis of the system dynamic process.

#### Components of farmers’ sustainable livelihood resilience

The descriptive framework of farmers’ sustainable livelihood resilience is composed of three elements: livelihood risk, livelihood system, and livelihood resilience. It is a dynamic process in which individuals or families use their livelihood capital to develop their buffer capacity, self-organization capacity, and learning capacity to better deal with uncertainty and recover from shocks, thereby maintaining livelihood stability and achieving sustainable development. Livelihood risks include all possible situations that farmers face in the process of maintaining their livelihood. These can arise from changes in the system’s external environment or the long-term accumulation of unstable factors within the system, including natural disasters, external policy changes, diseases, and education. The occurrence of livelihood risks impacts the different dimensions of the farmers’ livelihood system to varying degrees.

The farmers’ livelihood system is composed of all the elements that constitute or affect the farmers’ livelihood background, livelihood process, and livelihood means, and includes the farmers’ livelihood capital, which includes the farmers’ natural, physical, human, social, and financial resources. The farmers’ livelihood system is also affected by both the external environment and its internal structure, which we term external disturbances and internal disturbances, respectively. External disturbances refer to the influence of natural conditions or social backgrounds on farmers’ livelihoods and emphasize the connections and interactions with the outside world. Internal disturbances refer to the influence of the farmers’ labor force composition or income sources and emphasize the farmers’ livelihood structure.

Farmers’ livelihood resilience is reflected in their livelihood activities. Livelihood resilience maximizes their buffer against internal and external impacts, maintains their livelihood structure, and efficiently transforms their livelihoods in response to a variety of risks. It reflects the ability of farmers to maintain stable long-term development under various scenarios and is a means by which farmers’ livelihood systems cope with livelihood risks. Farmers’ livelihood resilience includes three dimensions: buffer capacity, self-organization capacity, and learning capacity. Buffer capacity refers to the ability of farmers to maintain their own functional attributes and organizational structure in response to internal and external disturbances, and can be understood as the first line of defense for farmers’ livelihood systems in the face of disturbances. Self-organization capacity refers to the ability of farmers to establish flexible communication and mutual assistance networks with the outside world, as well as their ability to integrate with the local economic, social, and institutional environments. When disturbances affect the farmers’ livelihood system, self-organization can mitigate the changes to the system caused by the disturbances. Learning capacity refers not only to the ability to acquire knowledge or skills but also to the ability of individual farmers and society members to exchange new knowledge and innovative production skills, thereby enhancing the farmers’ livelihoods. We argue that while these three dimensions jointly constitute the livelihood resilience of farmers, they also interact with each other, and the absence of any one of them affects the development of the other two as a result of system imbalance.

#### Descriptive framework of farmers’ sustainable livelihood resilience

Based on the above description of the key elements, the framework for describing the resilience of farmers’ livelihood constructed in this paper can be regarded as a composite system containing two major aspects: the natural environment and human society. In this system, with the rapid growth of population due to technological progress and productivity development, farmers face a complex and changing external environment in their production and life, and the resulting livelihood risks will put tremendous pressure on farmers’ livelihoods and directly affect their livelihood systems (Fig. 2a), causing significant changes in the stock of their internal livelihood capital. In order to adapt and maintain the needs of survival and development, the livelihood system of farmers drives the livelihood resilience of farmers by combining the livelihood capital they possess to make them respond (Fig. 2b). The livelihood resilience needs to be driven by a combination of internal buffering capacity, self-organizing capacity, and learning capacity, so that the livelihood system and livelihood risks can maintain their original state or transform into a new state under the effect of resilience (Fig. 2c). It is necessary to actively guide farm households to allocate livelihood assets rationally and improve livelihood resilience through the government’s macro-control policies, and it will lead to the poverty problem of farmers if reasonable guidance measures are not taken. Overall, the three main links interact with each other to form a dynamic system to achieve a virtuous cycle and sustainable development of farmers’ livelihoods. In addition, from a local perspective, the livelihood system and the livelihood resilience of farmers influence each other (Fig. 2d). First, resilience is determined by internal factors related to the livelihood system. Internal changes to the livelihood system as a result of shocks will inevitably lead to changes in resilience. However, because resilience represents the ability to deal with risks, it can, to some extent, control these internal changes. From a macro perspective, the farmers’ livelihood system is constantly evolving in response to risks and threats based on the farmers’ livelihood resilience, which moves through various stages of development until it eventually arrives at a stable ideal state.

### Measuring farmers’ sustainable livelihood resilience

The scientific measurement of farmers’ sustainable livelihood resilience is an important element of the application of the concept of resilience in the context of farmers’ livelihoods. Therefore, we constructed a resilience index in relation to farmers’ sustainable livelihoods using a cloud-model-based multi-level fuzzy comprehensive evaluation model to accurately measure the sustainable livelihood resilience of farmers. This model provides a reference point for poverty alleviation and the sustainable development of farmers’ livelihoods.

#### Farmers’ sustainable livelihood resilience index

Guided by the abovementioned descriptive framework of sustainable livelihood resilience, combined with the features of the study area, data availability, and the principles of indicator representation and scientificity, and using poverty registration data and results and trends from related studies to select relevant farmers’ livelihood resilience indicators (Zhang et al., 2020; Speranza et al., 2014; Wen et al., 2018; Liu et al., 2020; Li et al., 2022; Fachrista and Suryantini, 2019), we constructed an index measuring farmers’ sustainable livelihood resilience based on the three dimensions of buffer capacity, self-organization capacity, and learning capacity, as shown in Table 1.

**Table 1:** Index measuring farmers’ sustainable livelihood resilience.

Dimension	Indicator	Sub-indicator	Description
Buffer capacity	Natural capital	Terrain factor (−) (0.035)	Average slope, elevation, and undulation of administrative villages
		Environmental factor (0.046)	Green area (+)
			Drainage density (−)
		Agricultural land area per capita (+) (0.014)	(Farmland, forest, country, animal husbandry, water land area) mu/person
	Human capital	Total workforce score (+) (0.063)	Adult labor force/total headcount. Skilled labor force = 1, ordinary labor force = 0.66, weak labor force or semi-labor force = 0.33, other = 0
		Health status (+) (0.069)	Healthy population/total population. Healthy = 1, chronic disease = 0.5, serious illness or disability = 0
	Physical capital	Grade of dangerous house (+) (0.034)	A grade = 1, B grade = 0.75, C grade = 0.5, D grade = 0.25
	Financial capital	Household fixed assets (+) (0.037)	The number of commonly used fixed assets owned by farmers, with 1 point for each item owned
		Per capita annual net income (+) (0.051)	Annual net household income/total number of people
Self-organization capacity	Public service	Is there a passenger shuttle? (+) (0.023)	Yes = 1, no = 0
		Accessibility of educational facilities (+) (0.047)	Accessibility from the administrative village to all educational facilities within the county
		Accessibility of medical facilities (+) (0.048)	Accessibility from the administrative village to all health-care facilities within the county
	Social help	Social relationship (+) (0.042)	Family ethnic background. Non-minority = 1, minority = 0.66, sparsely populated minority = 0.33, ethnic groups with cross-stage development = –0.16 (−0.16)
		Whether a member of rural cooperatives (+) (0.047)	Joined = 1, not joined = 0
	Life security	Social security participation rate (+) (0.047)	Number of participants/total number of people
		Pension insurance participation rate (+) (0.034)	Number of participants/total number of people
		Critical illness insurance participation rate (+) (0.005)	Number of participants/total number of people
Learning capacity	Learning path	Number of village information officers (+) (0.041)	Number
		Whether the village has college students village officials (+) (0.063)	Yes = 1, no = 0
		Number of professional cooperatives in administrative villages (+) (0.043)	Number
	Means of livelihood	Percentage of income from agricultural production and operation (−) (0.05)	Agricultural production and operation income/total income
		Number of migrant workers (+) (0.044)	Number of migrant workers/total number of people
	Cultural reserve	Education status (+) (0.023)	Adult workforce total education/total number of people. Illiterate = 0, primary school = 0.25, junior high school = 0.5, high school or technical secondary school = 0.75, undergraduate or junior college = 1
		Political status (+) (0.028)	Number of non-masses/total number of people
		Mandarin prevalence (+) (0.066)	Number of people who can speak Mandarin/total number of people

Material in brackets indicates the sign and weight of each indicator.

Buffer capacity is the ability to resist livelihood pressure or disturbances by increasing one’s resource endowment, which includes five types of livelihood capital: natural, human, social, financial, and physical capital (Liu et al., 2020). Because of limited data availability, in this study, we evaluated buffer capacity based on four of the abovementioned sub-dimensions, natural, human, financial, and physical capital. The higher the level of farmers’ natural, human, financial, and physical capital, the greater their buffer capacity. Specifically, natural capital is represented by topographical factors, environmental factors, and per capita agricultural land area, human capital is represented by the total labor force and its health status, physical capital is represented by the grade of dangerous house, and financial capital is represented by household fixed assets and per capita annual net income.

Self-organization capacity is reflected in institutional policies, social organizations, and rural economic cooperation organizations (Lecegui et al., 2022; Fuchs, 2004). In this study, we divided self-organization capacity into three sub-dimensions: public services, social assistance, and living security, which represented the basic public service level in the administrative village where the farmer resided, the farmer’s social network, and social welfare indicators, respectively. The availability of passenger shuttles, accessibility of educational facilities, and accessibility of medical facilities were indicators of farmers’ ability to access public services. The extent of their social relationships measured the farmers’ levels of adaptation to society and inter-ethnic contact, and membership of rural cooperatives measured farmers’ access to development opportunities and degree of sharing of their own resources. These two indicators reflected the extent to which farmers could obtain community assistance. The extent of participation in social security, endowment insurance, and major illness insurance reflected the degree of maintenance and protection of farmers’ livelihoods by social system policies.

In terms of learning capacity, farmers with strong information channels, high production efficiency, and high levels of education and cultural literacy tend to have a greater learning capacity and are more able to minimize risks and resist shocks (Cooper and Wheeler, 2015). In this study, we divided learning capacity into three sub-dimensions: learning means, means of livelihood, and cultural reserves. The number of village information officers, whether there are college students in the village, and the number of professional cooperatives in administrative villages indicate opportunities for learning and communication, the proportion of agricultural production and operation income and the number of migrant workers indicate the ability of farmers to restore their livelihoods if their livelihoods are disturbed, and education status, political affiliation, and Mandarin prevalence indicate the level of access to information, policy awareness, and learning capacity, respectively.

#### Cloud-model-based multi-level fuzzy comprehensive evaluation model

In this study, the cloud-model-based multi-level fuzzy comprehensive evaluation model was used to measure buffer capacity, self-organization capacity, and learning capacity, so as to obtain the critical degree of different evaluation dimensions. The integration of the cloud model improved the objectivity of the results. The main process was as follows.

Using the abovementioned sustainable livelihood resilience index, the entropy weight method was used to obtain the weight of each index item, and the formulas are shown in formulas (1)–(3). Then, a judgment matrix was established to evaluate the relative importance of each index item. A nine-point Likert-type scale was used to measure the importance of each indicator, where 1, 3, 5, 7, and 9 represented “Unimportant,” “Slightly important,” “Moderately important,” “More important,” and “Very important,” respectively.

1 $$\begin{array}{*{20}{c}} {e_j = - \frac{1}{{{\rm {ln}}k}}\mathop {\sum }\limits_{i = 1}^k p_{ij}\ln ( {p_{ij}} )} \end{array}$$

2 $$\begin{array}{*{20}{c}} {w_j = \frac{{1 \,-\, e_j}}{{\mathop {\sum }\nolimits_{j = 1}^n 1 - e_j}}} \end{array}$$

The weight set of each indicator is obtained as

3 $$W = ( {w_1,w_2,\, \ldots ,w_j} )$$

where *e*_*j*_ is the entropy value of index *j*, *p*_*ij*_ is the standardized value of index *j* of farmer *i*, and *w*_*j*_ is the weight of index *j*.

Having determined the weight set and the judgment set, we carried out a fuzzy comprehensive evaluation. The cloud model was introduced to reduce the errors caused by random or accidental factors and improve the objectivity of the evaluation (DfID, 1999). In the cloud model, *X* is the normal set, *X* = {*x*} is the domain, and *T* is the language value associated with *X*. Fuzzy set *A* in domain *X* refers to *A* random number *A*(*x*) with a stable tendency for any element *x*, which is called the membership degree of *x* to *A*. The mathematical characteristics of the cloud model can be expressed in terms of expectation (Ex), entropy (En), and super-entropy (He). Using these three variables, fuzziness and randomness can be fully integrated to constitute a mapping of qualitative and quantitative to each other, providing a powerful means to combine qualitative and quantitative information processing.

We converted the five importance scales into cloud model importance scales, *E*_1_ = (Ex_1_, En_1_, He_1_)；*E*_2_ = (Ex_2_, En_2_, He_2_)；*E*_3_ = (Ex_3_, En_3_, He_3_)；*E*_4_ = (Ex_4_, En_*4*_, He_4_)；*E*_5_ = (Ex_5_, En_5_, He_5_), where Ex_*i*_ (*i* = 1, 2, 3, 4, 5) is the expectation, En_*i*_ (*i* = 1, 2, 3, 4, 5) is the judgment entropy, and He_*i*_ (*i* = 1, 2, 3, 4, 5) is the super-entropy. The fuzzy evaluation matrix *V* was obtained by inviting several experts to score the importance of each factor and entering the results into the cloud model, as shown in formulas (4)–(7):

4 $$V = \left( {V_1,V_2, \ldots ,V_m} \right) = \left[ {\begin{array}{*{20}{c}} {C_{11},C_{12}, \ldots ,C_{1k}} \\ {C_{21},C_{22}, \ldots ,C_{2k}} \\ \ldots \\ {C_{m1},C_{m2}, \ldots ,C_{mk}} \end{array}} \right] = \left[ {\begin{array}{*{20}{c}} {\left( {{\rm {Ex}}_{11},{\rm {En}}_{11},{\rm {He}}_{11}} \right), \ldots ,\left( {{\rm {Ex}}_{1k},{\rm {En}}_{1k},{\rm {He}}_{1k}} \right)} \\ {\left( {{\rm {Ex}}_{21},{\rm {En}}_{21},{\rm {He}}_{21}} \right), \ldots ,\left( {{\rm {Ex}}_{2k},{\rm {En}}_{2k},{\rm {He}}_{2k}} \right)} \\ \ldots \\ {\left( {{\rm {Ex}}_{m1},{\rm {En}}_{m1},{\rm {He}}_{m1}} \right), \ldots ,\left( {{\rm {Ex}}_{mk},{\rm {En}}_{mk},{\rm {He}}_{mk}} \right)} \end{array}} \right]$$

Thereinto:

5 $${\rm {Ex}} = \left( {C_{{\rm {min}}} + C_{{\rm {max}}}} \right)/2$$

6 $${\rm {En}} = \sqrt {\frac{\pi }{2}} \cdot {\rm {abs}}\left( {C_m} \right)$$

7 $${\rm {He}} = \sqrt {\left( {{\rm {abs}}\left( {C_m} \right)} \right)^2 - {\rm {Ex}}^2}$$

*C*_min_ and *C*_max_ are bilateral constraint relationships in the cloud model, *m* is the number of indicators participating in the evaluation, *k* is the number of experts participating in the evaluation, abs(*C*_*m*_) is the variance of data in the *m*th row, and Ex, En, and He are the evaluation expectation, entropy, and super-entropy, respectively, of the cloud model.

Through the fuzzy comprehensive evaluation results, the importance of each evaluation index to the evaluation object can be further judged. Based on previous studies (Cheng et al., 2020), the optimal level of evaluation parameters is Ex > En > He. To obtain a quantified result for the subsequent comprehensive evaluation and analysis, formula 8 is defined to represent the final fuzzy comprehensive evaluation score of each index:

8 $$P = \left( {{\rm {Ex}} + 0.25{\rm {En}} + 0.05{\rm {He}}} \right)\, * \,W$$

*P* is the final fuzzy comprehensive evaluation score of each index, Ex is expectation, En is entropy, He is super-entropy, and *W* is weight set.

To obtain the final resilience evaluation results, the final score for the fuzzy comprehensive evaluation of each indicator was used as the indicator weight, and the actual data in the case study are weighted and summed to quantify the sustainable livelihood resilience of each farmer. Before the comprehensive evaluation, the indicators were standardized to eliminate dimensional differences. The formulas are shown in formulas (9)–(12):

9 $${\rm {Re}} = \mathop {\sum }\limits_{i = 1}^m x_{i^ {\,\displaystyle{*}\,} }r_{i{\rm {Re}}}$$

10 $${\rm {Ad}} = \mathop {\sum }\limits_{i = 1}^m x_{i^ {\,\displaystyle{*}\,} }r_{i{\rm {Ad}}}$$

11 $${\rm {Tr}} = \mathop {\sum }\limits_{i = 1}^m x_{i^ {\,\displaystyle{*}\,} }r_{i{\rm {Tr}}}$$

12 $$R = {\rm {Re}}_{\rm {s}}\, * \,w_1 + {\rm {Ad}}_{\rm {s}}\, * \,w_2 + {\rm {Tr}}_{\rm {s}}\, *\, w_3$$

Re, Ad, and Tr are buffer capacity, self-organization capacity, and learning capacity, respectively, Re_s_, Ad_s_, and Tr_s_ are the standardized results of the three dimensions, *R* is the sustainable livelihood resilience of farmer *i*, *x*_*i*_ is the value of index *x* of farmer *i*, and *w*_1_, *w*_2_, and *w*_3_ are of equal weight, that is, 0.333.

### Analysis of farmers’ sustainable livelihood resilience

#### Coupling coordination degree model of farmers’ sustainable livelihood resilience

Because the coupling degree can only reflect the degree of coupling between the system or internal elements numerically, it is difficult to clearly identify the synergistic relationship underlying the interaction. To further explain the interaction intensity and coordinated development degree among the internal buffer capacity, self-organization capacity, and learning capacity of farmers’ sustainable livelihood resilience, we used the coupling coordination degree model for expression and analysis. The coupling coordination degree model is mainly used to characterize the strength of the interaction between multiple systems, and the coupling coordination degree can measure the degree of mutual promotion or hindrance of the internal elements of the system (Hong et al., 2022). The formula is as follows:

13 $$C_n^\prime = \left[ {\frac{{U1 \,*\, U2 \,*\, \ldots \,*\, Un}}{{\left( {U1 + U2 + \ldots + Un} \right)/n}}} \right]^{\frac{1}{n}}$$

14 $$D_{{\rm {ere}}} = \sqrt {C \,*\, T}$$

*n* is the number of subsystems, *Un* is the subsystem score, *C* is the system coupling degree score, and *T* is the comprehensive value of the system.

In this study, the coupling coordination degree describes whether the buffer capacity, self-organization capacity, and learning capacity of farmers have a good level and interaction among sustainable livelihood resilience. The degree of coupling and coordination among the three dimensions and their changing trends directly affected the level of internal coordinated development of farmers’ sustainable livelihood resilience in each administrative village. The coupling coordination degree results were used to classify the coupling coordination relationships among buffer capacity, self-organization capacity, and learning capacity using the classification criteria shown in Table 2.

**Table 2:** Classification of coupling coordination relationships.

Classification interval	Coupling coordination degree		Coupling coordination type	Explanation
0 < *D* ≤ 0.25	Low	Strong obstruction type		There is a strong mutually hindering relationship between the buffer, self-organization, and learning capacity dimensions, forming a development situation with serious internal constraints of resilience
0.25 < *D* ≤ 0.5	Medium	Weak obstruction type		There is a weak mutually hindering relationship between the buffer, self-organization, and learning capacity dimensions, forming a development situation with internal constraints of resilience
0.5 < *D* ≤ 0.75	Medium	Weak promotion type		There is a weak mutually supportive relationship between the buffer, self-organization, and learning capacity dimensions, forming a development situation with internal coordination of resilience
0.75 < *D* ≤ 1	High	Strong promotion type		There is a strong mutually supportive relationship between the buffer, self-organization, and learning capacity dimensions, forming a development situation with strong internal coordination of resilience

#### Classification of farmers’ sustainable livelihood resilience types

With the aim of promoting the stable development of the farmers’ sustainable livelihood system, we constructed a decision tree that can be used to classify farmers’ sustainable livelihood resilience into various types to enable targeted countermeasures and suggestions for farmers with different sustainable livelihood resilience characteristics.

**Fig. 3:**
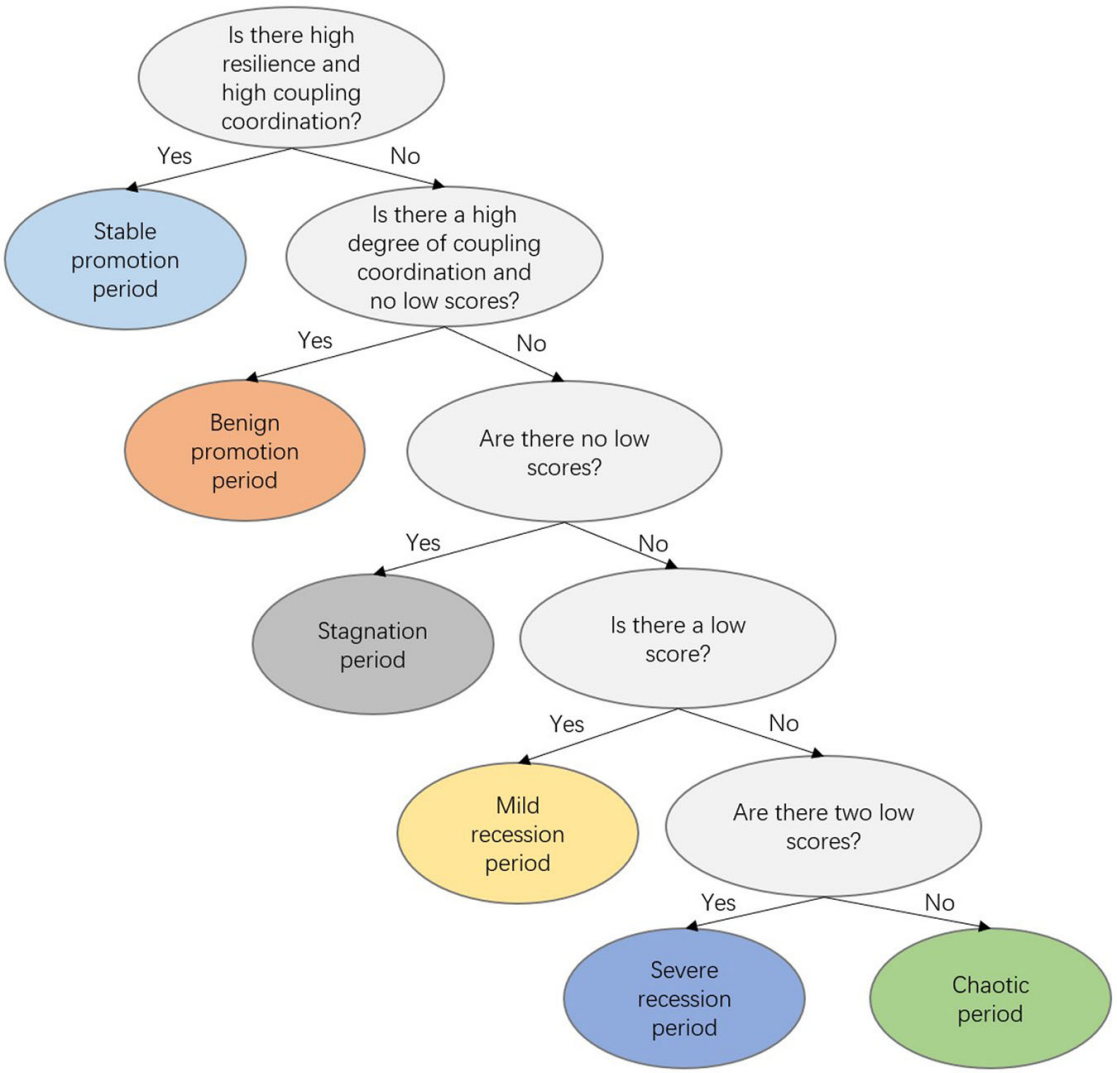
Decision tree for the classification of farmers’ sustainable livelihood resilience types. The figure shows the classification rules and classification results for classifying the six state types of sustainable livelihood resilience of farmers using the decision tree approach.

The decision tree method is commonly used in machine learning because it can analyze large amounts of data in a relatively short period of time. The decision tree algorithm we constructed is easy to understand and has strong interpretability and visualization, and relatively simple classification rules. The top-down recursive method is used to compare attribute values at the internal nodes of the decision tree, and then to judge the branches down from the node based on different attribute values to reach a conclusion at the leaf node of the decision tree. The construction of a decision tree involves the following steps: (1) Feature selection on the data set to form a training set; (2) Training on the training set to generate an initial decision tree; (3) Pruning of the initial decision tree; and (4) Extracting classification rules based on the final decision tree. In this study, the classification rules were obtained based on the results of various scores of farmers in the resilience evaluation and coupling coordination degree, as shown in Fig. 3 and Table 3.

**Table 3:** Definitions of farmers’ sustainable livelihood resilience types.

Period	Explanation
Stable promotion period	With strong and stable anti-risk ability, the elements of resilience can promote each other and achieve sustainable livelihood
Benign promotion period	With strong anti-risk ability, the elements of resilience promote each other to form a virtuous circle, and gradually transition to a period of stable improvement
Stagnation period	It has the strong anti-risk ability. Although the elements of resilience have no obvious defects, they are separated from each other, and the overall development has fallen into a bottleneck. Although the status quo can be maintained, it is not conducive to achieving long-term sustainable livelihoods
Mild recession period	The ability to resist risks is poor, and there is a defect in one of the elements that make up resilience, which hinders the improvement of resilience, and the livelihood capacity begins to decline
Severe recession period	The ability to resist risks is poor, two of the elements that make up resilience are flawed, development is severely hindered, and livelihoods are rapidly declining
Chaotic period	Completely exposed to risk, inadequate capacity in all aspects, and sink deeper and deeper into a vicious circle

## Results and analysis

### Farmers’ sustainable livelihood resilience analysis

**Fig. 4:**
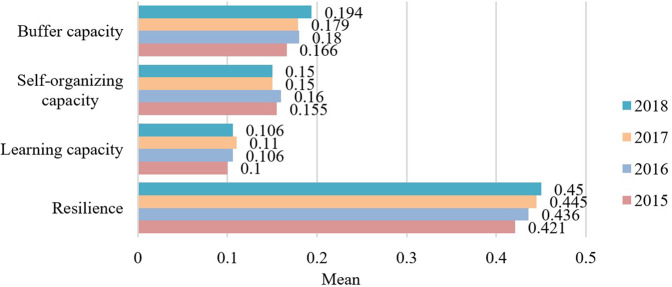
Spatial distribution of sustainable livelihood resilience of farmers in Fugong County from 2015 to 2018. The figure shows the change in the mean values of the sustainable livelihood resilience of farm households and their internal buffer capacity, self-organization capacity, and learning capacity dimensions from 2015 to 2018, with larger values representing higher sustainable livelihood resilience of farm households.

**Fig. 5:**
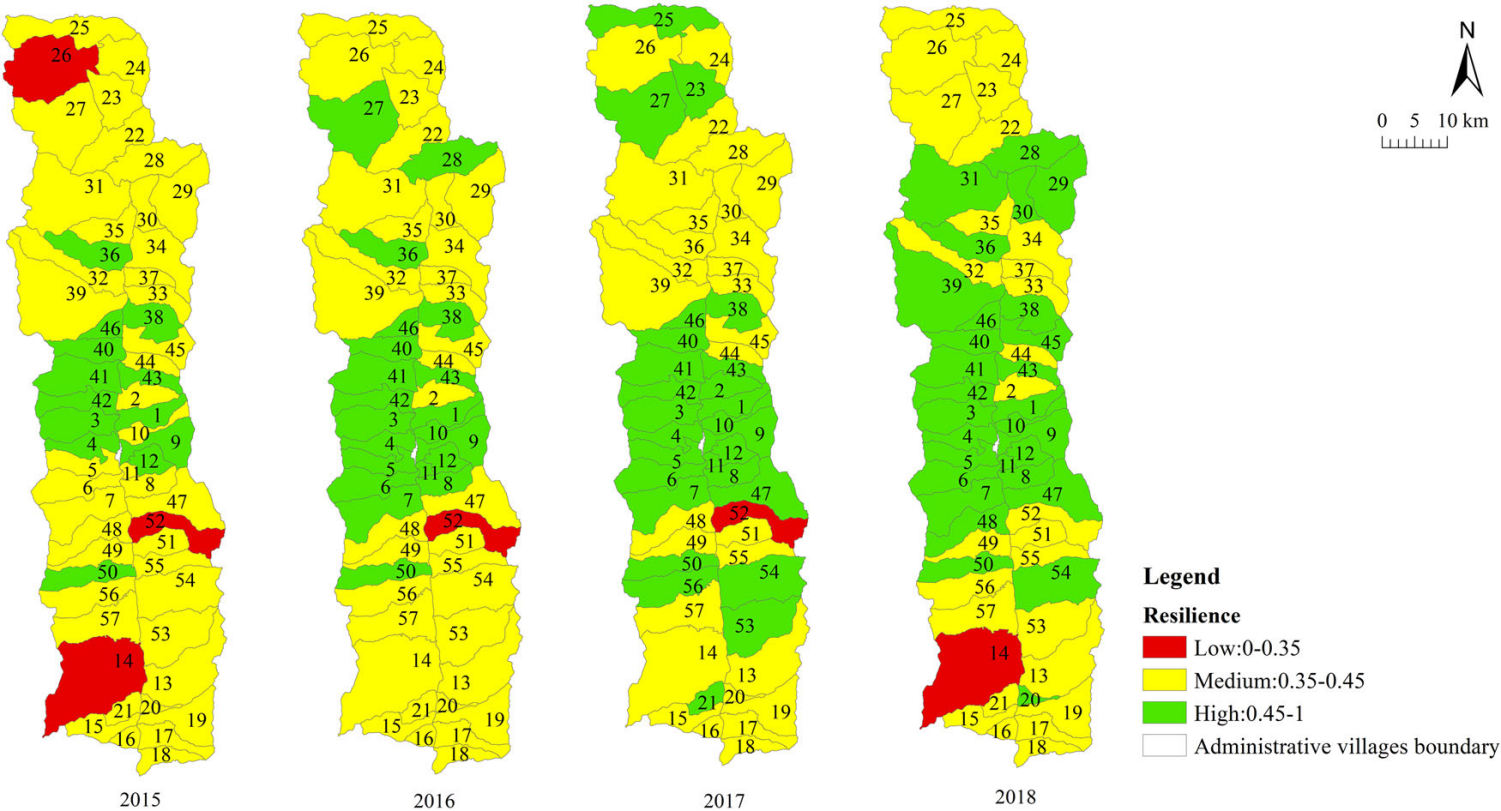
Village-scale spatial distribution of sustainable livelihood resilience of farmers in Fugong County from 2015 to 2018. The figure shows the spatial distribution of the results of the sustainable livelihood resilience of farm households obtained by the cloud-model-based multi-level fuzzy comprehensive evaluation model in each administrative village in the study area.

**Fig. 6:**
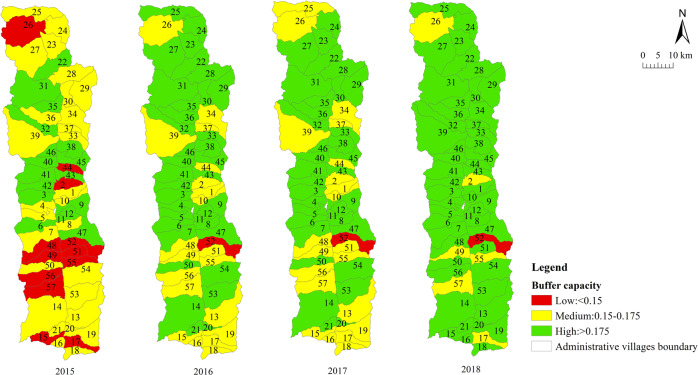
Village-scale spatial distribution of buffer capacity for sustainable livelihood resilience of farmers in Fugong County from 2015 to 2018. The figure shows the spatial distribution of the results of the internal buffer capacity dimension for sustainable livelihood resilience of farm households obtained by the cloud-model-based multi-level fuzzy comprehensive evaluation model in each administrative village in the study area.

**Fig. 7:**
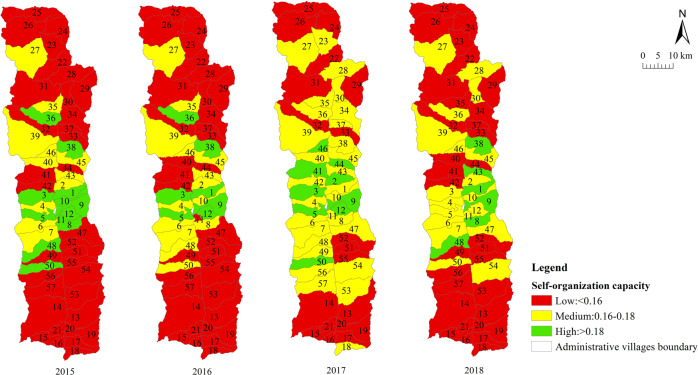
Village-scale spatial distribution of self-organization capability for sustainable livelihood resilience of farmers in Fugong County from 2015 to 2018. The figure shows the spatial distribution of the internal self-organization capacity dimension for sustainable livelihood resilience of farm households obtained by the cloud-model-based multi-level fuzzy comprehensive evaluation model in each administrative village in the study area.

**Fig. 8:**
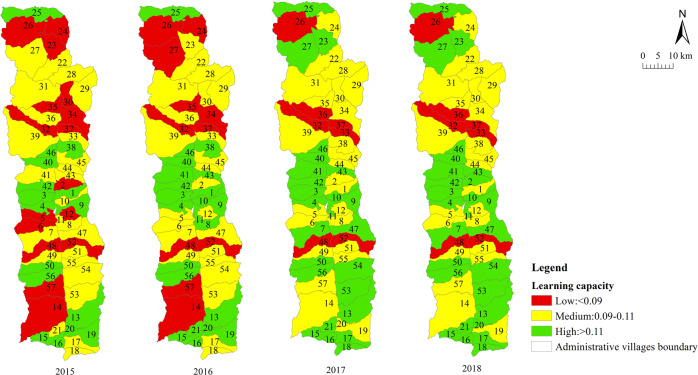
Village-scale spatial distribution of learning capability for sustainable livelihood resilience of farmers in Fugong County from 2015 to 2018. The figure shows the spatial distribution of the result of the internal learning capacity dimension for sustainable livelihood resilience of farm households obtained by the cloud-model-based multi-level fuzzy comprehensive evaluation model in each administrative village in the study area.

We measured the sustainable livelihood resilience of farmers in Fugong County from 2015 to 2018. The statistical results are summarized in Table 4 and the spatial distribution is displayed in Fig. 4. The results at the village scale are shown in Table 5 and the spatial distribution is displayed in Figs. 5–8.

**Table 4:** Sustainable livelihood resilience of farmers in Fugong County from 2015 to 2018.

		2015	2016	2017	2018
Resilience	Mean	0.421	0.436	0.445	0.45
	Standard deviation	0.059	0.055	0.058	0.06
Buffer capacity	Mean	0.166	0.18	0.179	0.194
	Standard deviation	0.029	0.026	0.026	0.025
Self-organization capacity	Mean	0.155	0.15	0.16	0.15
	Standard deviation	0.032	0.035	0.032	0.033
Learning capacity	Mean	0.1	0.106	0.11	0.106
	Standard deviation	0.03	0.027	0.028	0.031

**Table 5:** Village-scale sustainable livelihood resilience of farmers in Fugong County from 2015 to 2018.

	2015	2016	2017	2018
*Mean*				
Resilience	0.421	0.436	0.445	0.45
Buffer capacity	0.166	0.18	0.179	0.194
Self-organization capacity	0.153	0.148	0.157	0.148
Learning capacity	0.102	0.107	0.108	0.107
*Standard deviation*				
Resilience	0.04	0.038	0.041	0.042
Buffer capacity	0.016	0.014	0.015	0.014
Self-organization capacity	0.025	0.026	0.025	0.026
Learning capacity	0.018	0.016	0.017	0.018
*Top three*				
Resilience	Shangpa Village (0.51) Chisadi Village (0.496) Bajiaduo Village (0.487)	Shidi Village (0.515) Shangpa Village (0.502) Chisadi Village (0.492)	Chisadi Village (0.53) Shangpa Village (0.525) Shidi Village (0.521)	Chisadi Village (0.554) Shangpa Village (0.553) Lazhudi Village (0.524)
Buffer capacity	Buladi Village (0.210) Bajiaduo Village (0.204) Lumadeng Village (0.192)	Buladi Village (0.211) Chisadi Village (0.209) Lawu Village (0.204)	Buladi Village (0.21) Chisadi Village (0.209) Lawu Village (0.207)	Buladi Village (0.223) Chisadi Village (0.222) Lawu Village (0.218)
Self-organization capacity	Shuangmidi Village (0.189) Shangpa Village (0.188) Chisadi Village (0.187)	Ziguduo Village (0.189) Shangpa Village (0.187) Shidi Village (0.186)	Shangpa Village (0.203) Liwudi Village (0.195) Shuangmidi Village (0.191)	Shangpa Village (0.199) Chisadi Village (0.197) Lazhudi Village (0.195)
Learning capacity	Laomudeng Village (0.139) Tuoping Village (0.137) Shangpa Village (0.133)	Bula Village (0.151) Maji Village (0.14) Lamujia Village (0.133)	Bula Village (0.152) Maji Village (0.138) Miangu Village (0.135)	Laomudeng Village (0.15) Shangpa Village (0.142) Zhiziluo Village (0.141)
*Bottom three*				
Resilience	Mujiajia Village (0.333) Wawa Village (0.339) Dadake Village (0.345)	Dadake Village (0.341) Wawa Village (0.351) Mujiajia Village (0.363)	Dadake Village (0.346) Wawa Village (0.357) Mujiajia Village (0.368)	Wawa Village (0.358) Dadake Village (0.369) Puluo Village (0.372)
Buffer capacity	Dadake Village (0.129) Mujiajia Village (0.138) Dapuluo Village (0.139)	Dadake Village (0.141) Puluo Village (0.156) Dapuluo Village (0.158)	Dadake Village (0.139) Puluo Village (0.155) Ekeluo Village (0.156)	Dadake Village (0.164) Ekeluo Village (0.165) Puluo Village (0.171)
Self-organization capacity	Bula Village (0.082) Wawa Village (0.087) Puluo Village (0.106)	Wawa Village (0.076) Puluo Village (0.095) Bula Village (0.102)	Wawa Village (0.084) Puluo Village (0.103) Laomudeng Village (0.104)	Wawa Village (0.073) Bula Village (0.09) Puluo Village (0.092)
Learning capacity	Dadake Village (0.062) Zhiluo Village (0.072) Yaduo Village (0.074)	Dadake Village (0.065) Weidu Village (0.075) Mujiajia Village (0.076)	Dadake Village (0.064) Weidu Village (0.075) Mujiajia Village (0.076)	Dadake Village (0.064) Yaduo Village (0.076) Mujiajia Village (0.077)

As can be seen from Table 4 and Fig. 4, farmers’ sustainable livelihood resilience showed a relatively weak increasing trend from 2015 to 2018, during which buffer capacity increased significantly, while self-organization capacity and learning capacity remained relatively stable. This is because income is not the only evaluation criterion for resilience. To improve resilience, it is necessary to target all aspects of farmers’ livelihoods. In addition, the standard deviations of the four statistical indicators all showed a gradually increasing trend, indicating that the imbalance between levels of development is widespread and increasing.

As can be seen from Table 5 and Figs. 5–8, the resilience measurement results of villages as a statistical unit, from the overall value of the 4 years, the average buffer capacity, self-organization capacity, and learning capacity of villages in Fugong County from 2015 to 2018 showed a slight increase or fluctuated trend, with little overall change. It can be further found in Figs. 5–8 that, as time goes on, both the resilience of farmers’ livelihood in Fugong County and the following three dimensions show a gradually increasing trend, indicating that the resilience of each administrative village has been improved to varying degrees. However, some areas remained at a low level of development at the end of the period. Looking at the overall distribution, the situation in the central region was better than that in the northern and southern regions, and the administrative villages with low resilience levels were mainly distributed in the southwestern and northwestern regions. At the administrative village level, the resilience levels of Shidi Village, Shangpa Village, and Chisadi Village were all relatively high from 2015 to 2018, while those of Mujiajia Village, Wawa Village, and Dadake Village were relatively low. In terms of buffer capacity, Buladi Village was the highest, and Dadake Village was the lowest, in terms of self-organization capacity, Shangpa Village and Chisadi Village maintained a relatively high level, while Wawa Village, Puluo Village, and Bula Village remained at a low level, and in terms of learning capacity, Bula Village, Maji Village, Laomudeng Village, and Shangpa Village remained at a relatively high level, while Dadake Village, Weidu Village, Mujiajia Village, and Yaduo Village remained at a low level.

### Internal coupling coordination degree of farmers’ sustainable livelihood resilience

**Fig. 9:**
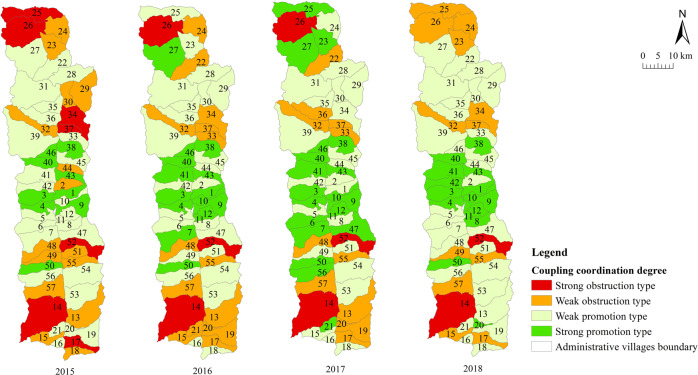
Distribution of the coupling coordination degree of sustainable livelihood resilience of farmers in Fugong County from 2015 to 2018. The figure shows the spatial distribution of the four types of coupling coordination in each administrative village in the study area according to the results of the coupling coordination model.

To explore the coupling and coordinated development relationships among the internal buffer capacity, self-organization capacity, and learning capacity dimensions of farmers’ sustainable livelihood resilience, we measured the internal coupling coordination degree of farmers’ sustainable livelihood resilience in Fugong County from 2015 to 2018. The statistical results are shown in Table 6 and the spatial distribution of coupling coordination degree types at the administrative village level is shown in Fig. 9.

**Table 6:** Statistical results of the coupling coordination degree of sustainable livelihood resilience of farmers in Fugong County from 2015 to 2018.

	2015	2016	2017	2018
Mean	0.404	0.42	0.436	0.471
Standard deviation	0.064	0.059	0.062	0.066
The top three villages in household average coupling coordination	Shangpa Village (0.541) Chisadi Village (0.538) Shidi Village (0.494)	Shidi Village (0.503) Shangpa Village (0.486) Buladi Village (0.473)	Buladi Village (0.518) Shidi Village (0.510) Shangpa Village (0.508)	Shangpa Village (0.541) Chisadi Village (0.538) Lazhudi Village (0.515)
The bottom three villages in household average coupling coordination	Dadake Village (0.317) Mujiajia Village (0.318) Wawa Village (0.319)	Dadake Village (0.319) Wawa Village (0.322) Mujiajia Village (0.341)	Dadake Village (0.323) Mujiajia Village (0.332) Wawa Village (0.347)	Wawa Village (0.321) Dadake Village (0.339) Mujiajia Village (0.354)

It can be seen from Table 6 that the overall resilience coupling coordination level in Fugong County gradually increased from 2015 to 2018, and the villages with the highest levels of coupling coordination included Shangpa Village, Chisadi Village, Shidi Village, Buladi Village, and Lazhudi Village, while those with the lowest levels included Dadake Village, Mujiajia Village, and Wawa Village. It can be seen from Fig. 9 that the coupling coordination degree of farmers’ sustainable livelihood resilience was generally higher in the central region and lower in the northern and southern regions, with a general trend of widespread dispersion and low aggregation. Moreover, the number of administrative villages in Fugong County with weak and strong coupling coordination degrees of promotion gradually increased over time. The strong promotion villages were mainly concentrated in the central region, and the overall showed a trend of gradually spread to the north and the south. Combining these results with those of our analysis of resilience, it can be seen that the three dimensions of buffer capacity, self-organization capacity, and learning capacity of farmers in this type of administrative village complement each other, synergistically promoting the sustainable development of both themselves and others and maintaining each other at a high level, thereby further promoting the overall improvement of farmers’ sustainable livelihood resilience. However, the coupling coordination degree of the administrative villages in the northern and southern border regions is generally low, and the weak promotion and weak obstruction administrative villages are mainly distributed on the north and south sides and spread to the edge. It can also be seen from the earlier results regarding the three dimensions that self-organization capacity has not improved as much as the other two dimensions, resulting in insufficient coordinated development among the three dimensions. If there is no improvement in this dimension, it will inevitably affect the coordinated development of farmers’ sustainable livelihood resilience. Strong obstruction administrative villages are mainly distributed in the northern and southwestern regions of the county. The degree of coupling coordination in these regions remains at a low level, and thus coordinated development is yet to occur. The absence of one dimension hinders the development of the other two, thereby reducing farmers’ overall sustainable livelihood resilience and creating a vicious circle.

It can be seen that buffer capacity, self-organization capacity, and learning capacity influence each other, interact with each other, and develop collaboratively, and the lack of any one dimension will affect the development of farmers’ sustainable livelihood resilience. To improve the level of internal coordinated development of farmers’ sustainable livelihood resilience, it is necessary to promote the coordinated development of all elements of rural society including infrastructure, economic development, public services and social governance from the multi-field, multi-subject and multi-resource collaboration. Overall planning and coordination should be strengthened in an effort to achieve ongoing coordinated development among the three internal dimensions of resilience to continuously improve the system’s ability to respond to external risks and shocks and ultimately achieve a state of sustainable development.

### The development of farmers’ sustainable livelihood resilience

**Fig. 10:**
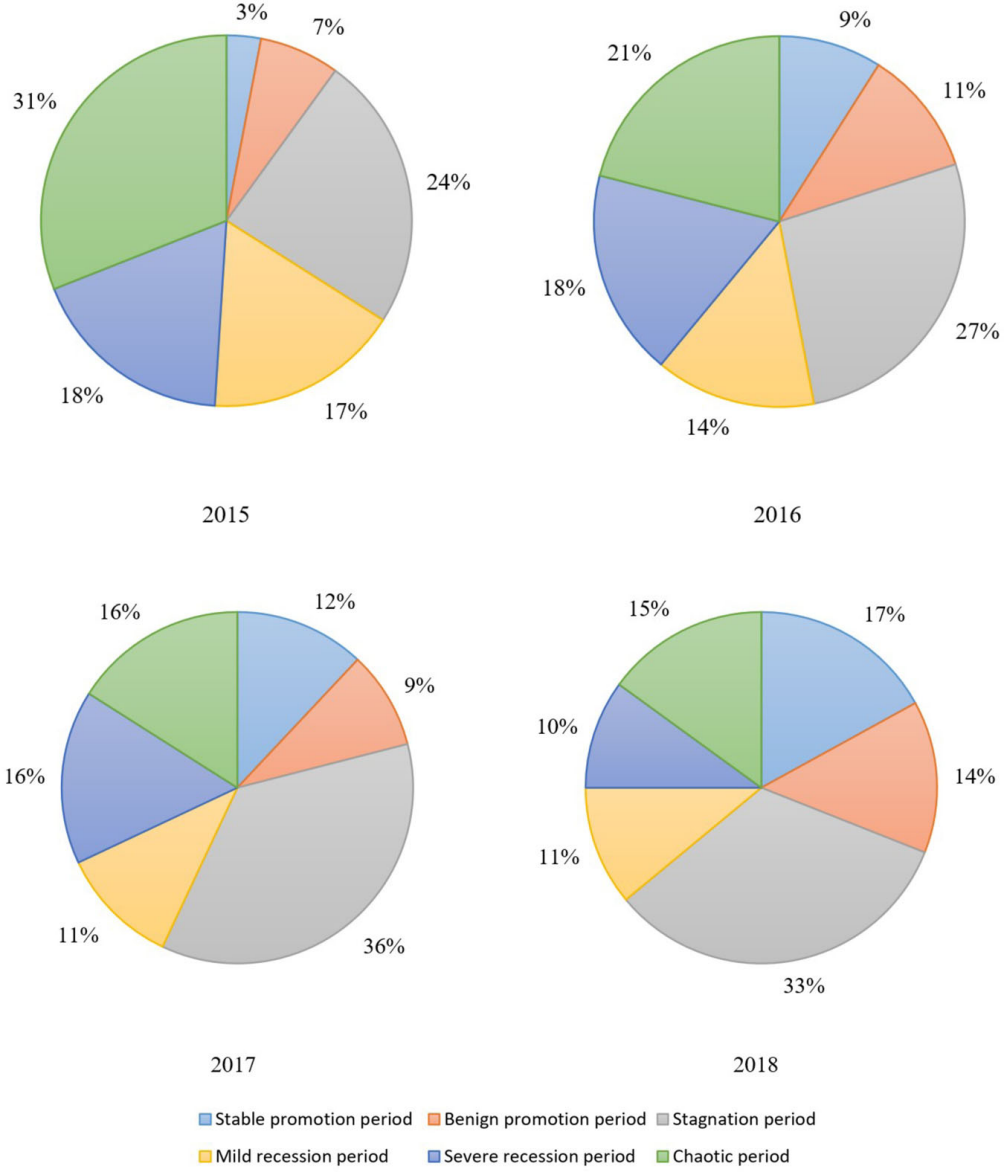
Distribution of farmers’ sustainable livelihood resilience state types in Fugong County from 2015 to 2018. The figure shows the percentage of six different types of sustainable livelihood resilience states of farm households in Fugong County classified by decision tree method during the four years from 2015 to 2018. Among them, a larger proportion of the promotion period indicates a better sustainable livelihood resilience development, while a larger proportion of the stagnation, recession, and chaotic periods indicates a poor sustainable livelihood resilience development.

We divided the sustainable livelihood resilience development status of farmers in the study area into six categories, as shown in Fig. 10. It can be seen that although the proportion of farmers in the stable promotion category was relatively small, it increased from 3% in 2015 to 17% in 2018. The proportion of farmers in the benign promotion category fluctuated over the period but grew from 7% in 2015 to 14% in 2018. Farmers in these two categories have relatively rich resource endowments and relatively diverse means of sustaining their livelihood, and can better resist internal and external disturbances, adapt to external shocks, and undertake rapid transformation. Farmers in the stagnation category accounted for the largest proportion, reaching a maximum of 36% in 2017, indicating that a large number of farmers in Fugong County have overcome their livelihood difficulties but failed to build strong livelihood resilience. They lack an endogenous driving force for development, and thus further guidance is needed to achieve consolidation or transformation to improve their livelihood resilience and enable them to achieve sustainable development. Farmers in the mild recession category and severe recession category accounted for relatively large proportions overall, but showed a gradual downward trend, decreasing by 6% and 8%, respectively, over the 4-year period. In these categories, there are obvious deficiencies in specific elements, which affects their overall resilience level and further hinders these farmers’ livelihood development. Thus, for these farmers, it is necessary to precisely identify their shortcomings, prescribe the right solutions, and establish a stable livelihood resilience state as quickly as possible. Farmers in the chaotic category accounted for 31% of the total in 2015, but this had declined to 15% by 2018. All aspects of these farmers’ sustainable livelihood resilience were at a low level, and they were significantly threatened by various livelihood risks. The mutual hindrance among the internal factors relating to their resilience made the livelihoods of these farmers increasingly stressed and created a vicious circle. Farmers in this category need comprehensive assistance and guidance to enable them to overcome their difficulties and gradually develop their sustainable livelihood resilience.

Overall, the results showed that from 2015 to 2018, the number of farmers in Fugong County making progress in terms of sustainable livelihood resilience increased year by year, indicating that the local assistance program has achieved tangible results. However, the number of rural households in the stagnation and chaotic categories continue to account for a large proportion of households, indicating that there is still a large number of rural households with poor development capacity that continue to live in poverty. Thus, it is necessary to further focus on key areas and focus on tackling tough problems, so that it can step into a benign path of stable development.

## Discussion and conclusion

In this study, we used resilience theory combined with the traditional sustainable livelihood analysis framework to construct a descriptive framework of farmers’ sustainable livelihood resilience. Then, we constructed an index of farmers’ sustainable livelihood resilience and a measurement model using a cloud-model-based fuzzy comprehensive evaluation model using the three internal resilience dimensions of buffer capacity, self-organization capacity, and learning capacity. Finally, taking Fugong County, Yunnan Province, China as the study area, we used coupling coordination degree, decision tree, and other methods of analysis to measure the internal synergy and development status of farmers’ sustainable livelihood resilience during the period from 2015 to 2018.

The results were as follows. Overall, the sustainable livelihood resilience of farmers in Fugong County in 2015, 2016, 2017, and 2018 was 0.421, 0.436, 0.445, and 0.450, respectively, indicating a gradual upward trend. Administrative villages with high resilience levels were mainly concentrated in the central region, those with medium resilience levels were mainly distributed in the northern and southern regions, and those with low resilience levels were mainly distributed in the southwestern and northwestern border regions. At the administrative village level, the villages with the highest overall levels of farmers’ sustainable livelihood resilience were Shangpa Village, Shidi Village, and Chisadi Village, while those with the lowest levels were Dadake Village, Wawa Village, and Mujiajia Village. Buladi Village had the highest buffer capacity, while Dadake Village has the lowest, Shangpa Village and Chisadi Village had the highest self-organization capacity, while Wawa Village, Puluo Village, and Bula Village had the lowest, and Laomudeng Village and Bula Village had the highest learning capacity, while Dadake Village and Weidu Village had the lowest. Thus, it can be seen that farmers’ sustainable livelihood resilience was unevenly distributed in terms of both space and time. The average coupling coordination degree of administrative villages in Fugong County in 2015, 2016, 2017, and 2018 was 0.404, 0.420, 0.436, and 0.471, respectively, once again indicating an upward trend. In terms of spatial distribution, the coupling coordination degree in the central region of Fugong County was significantly higher than that in the northern and southern regions, and lowest in the northern and southwestern regions. The administrative villages with the highest degree of coupling coordination were Shangpa Village, Chisadi Village, Shidi Village, Buladi Village, and Lazhudi Village, while those with the lowest degree were Dadake Village, Mujiajia Village, and Wawa Village. Therefore, the spatial distribution of the coordinated development level of farmers’ sustainable livelihood resilience is similar to that of its overall level, indicating that buffer capacity, self-organization capacity, and learning capacity influence each other, interact with each other, and develop collaboratively. The lack of any one of these three dimensions will affect the overall development of farmers’ sustainable livelihood resilience. Farmers’ sustainable livelihood resilience can be divided into six categories: stable promotion, benign promotion, stagnation, mild recession, severe recession, and chaotic period. During the period from 2015 to 2018, the proportion of farmers in the two promotion categories increased by 21%, in the two recession categories decreased by 14%, and in the stagnation and chaotic categories remained relatively high. The levels of farmers’ sustainable livelihood resilience and its coupled coordination provide important information for the government in relation to its efforts to formulate targeted assistance policies for different types of farmers, which will help poor areas gradually improve their sustainable livelihood resilience.

The results of this study contribute to farmer livelihood research in the field of sustainable development in the context of poverty alleviation and help to fill the gaps in international resilience theory research. In response to turbulent situations such as the current COVID-19 pandemic, our findings will help governments to target low-resilience groups, identify the factors necessary to promote sustainable livelihood resilience development in various poverty-stricken groups and provide a sound basis for rational, targeted decision-making. They also provide a scientific reference point for local governments in determining assistance goals and formulating appropriate development policies and support strategies based on regional characteristics, which is conducive to improving the livelihood structure of farmers, alleviating poverty, and promoting the long-term sustainable development of farmers’ livelihood resilience.

However, given the complexity of the resilience system, this study has some limitations. First, in this study, we only analyzed data from 2015 to 2018, which may miss the characteristics and changes of farmers’ sustainable livelihood resilience in certain time periods, especially during the period when the COVID-19 pandemic seriously disrupted the sustainable development of farmers’ livelihoods in the past three years. Thus, future studies should obtain data over a longer time frame, including more up-to-date data, to enable a more comprehensive analysis to be undertaken. Second, changes in the level of resilience in response to various influencing factors were not examined in detail because of space limitations. Thus, future studies should focus on changes in the level of resilience in response to a range of influencing factors in an effort to identify the underlying mechanisms and relationships among the various elements of the framework for farmers’ sustainable livelihood resilience, thereby enhancing both the theory and practical application of farmers’ sustainable livelihood resilience systems.

## Data Availability

The datasets generated during and/or analysed during the current study are available from the corresponding author on reasonable request.
